# 

**Published:** 1961-10

**Authors:** 


### MEDICAL JOURNAL OF THE SOUTH-WEST

*(Incorporating the Bristol Medico-Chirurgical Journal)*

Established 1883

VOL. 76 (iv) October 1961 NO. 282

### *Contents*

THE UNIVERSITY AND THE MEDICAL SCHOOL *Sir Philip Morris* 103

FUNGUS INFECTIONS OF THE SKIN IN BRISTOL AND THE WEST COUNTRY . . . . . . . *Mary P. English* 112

THE PROBLEM OF HEADACHE . . . . . *F. T. Page* 122

BRONCHOPNEUMONIA DUE TO *PASTEURELLA MULTOCIDA* (*SEPTICA*) . . . . . . *G. A. Hayes and G. Neale* 129

ANKYLOSING SPONDYLITIS WITH MITRAL STENOSIS *Clinico—Pathological Conference* 132

NOTES AND NEWS . . . . . . . . 143

APPOINTMENTS . . . . . . . . 144

NEWS FROM THE SOCIETIES . . . . . . . 146

*PUBLISHED QUARTERLY*

*ANNUAL SUBSCRIPTION* 16/- *POST FREE*

PUBLISHED UNDER THE AUSPICES OF THE
BRISTOL MEDICO-CHIRURGICAL SOCIETY

### *Editorial Committee*

Dr. J. E. Cates, Department of Medicine, Bristol Royal Infirmary. *Editor.*
Dr. N. J. Brown, Southmead Hospital, Bristol. *Assistant Editor.*
Dr. A. B. Raper, Department of Pathology, Bristol Royal Infirmary. *Assistant Editor*
Dr. J. Macrae, Ham Green Hospital.
Dr. R. C. Wofinden, Central Clinic, Bristol.
Mr. A. E. S. Roberts, Medical Library, University of Bristol. *Secretary.*

### *Local Correspondents*

*Bath* . . Mr. A. D. Bateman, 3 The Circus, Bath.
*Exeter* . . Dr. L. N. Jackson, Mount Jocelyn, Crediton, Devon.
*Gloucester* . Dr. R. F. Jarrett, 9 College Green, Gloucester.
*Plymouth* . Dr. C. R. Croft, Lexden, Hartley Av., Plymouth.
*Taunton* . Dr. M. McAlley, 1 The Crescent, Taunton.
*Torquay* . Dr. A. Robinson Thomas, 20 Devon Square, Newton Abbot, Devon.
*Truro* . . Dr. F. D. M. Hocking, St. Andrews, Perranporth, Cornwall.
*Yeovil* . . Mr. J. A. Vere Nicoll, Knapp House, Yeovil, Somerset.

### NOTICE TO CONTRIBUTORS

Original articles are invited, provided they have not been published elsewhere. Notes of forthcoming events, meetings held, degrees conferred, staff appointments, and other local news are welcomed. The editorial committee reserves the right to make literary corrections.

Illustrations should always be supplied ready for reproduction. All re-touchings or other operations required will be charged to the author. Line drawings should be drawn in Indian ink. We regret that we must ask authors to pay for making blocks, if this is necessary.

J. W. ARROWSMITH LTD., WINTERSTOKE ROAD,
BRISTOL.

### *Notes and News*

Professor R. Milnes Walker has been re-elected a member of the Council of the Royal College of Surgeons of England.

Professor A. I. Darling gave a Charles Tomes lecture on 21st July at the Royal College of Surgeons on the selective attack of caries on the dental enamel.

Professor J. M. Yoffey has been awarded a first prize and a silver medal for the film "The Lymphatic System" by the Film Committee of the Eighth International Congress of Haematology held in Tokyo in September 1960.

OBITUARY:

H. H. Carleton
J. J. Kempton
E. Scott White

### *Examination Results*

*M.R.C.P.:* K. R. Gough

### UNIVERSITY OF BRISTOL

*M.D.*

N. L. Browse

I. D. Fraser

Kathleen J. Harrison

T. Valaes

*Ph.D.:*

L. N. Payne
C. I. Phillips

*D.P.H.:*

M. R. Alderson

M. O. H. Gritley

J. M. Joshua

P. M. Lambert

Shirley A. Lewis

J. G. Meadows

C. P. B. Parry

A. J. Rowland

A. M. A. Saleem

M. J. Sanecki

Tay Za Aung

K. D. Young

Olga E. Zamenhof-Nietupska

*M.B., Ch.B.:*

SECOND CLASS HOUOURS

G. J. A. Brown (*with distinction in surgery*)

A. A. Brzechwa-Ajudkiewicz

Edith M. Creighton

B. H. Cummins

A. G. Morgan

J. A. Morphew

Violet A. Payne

Diana M. Young

PASS

D. W. R. Carr

A. M. Chrysaphinis

Marjorie A. Coutts

Aerona M. A. Crick

M. J. Crick

K. A. Day

A. B. O. Desalu

J. D. Egdell

I. F. Elliott

K. H. Gay

G. H. Gundy

C. P. Hallett

B. Hanstead

D. W. Hide

Eleanor J. A. Hughes

R. C. W. Hughes

N. Ikoku

W. A. Knight

J. M. McGarry

Vida V. Mungwira

A. S. Parker

R. F. Payne

M. F. Reed

R. R. Roy (*with distinction in public health*)

A. K. Smeeton

T. D. Waterhouse

M. R. Watts

### Appointments

Dr. W. A. Gillespie has been appointed to the newly-established part-time chair in clinical bacteriology at the University of Bristol.

Professor J. N. P. Davies, professor of pathology at Makerere College, has been appointed to the readership in morbid anatomy at the Postgraduate Medical School of London.

Dr. P. O. Nicholas has been appointed deputy M.O.H. Adwick-le-Street, Bentley-with-Arksey and Tickhill.

*F.R.C.P.:*

Dr. R. P. Warin
Dr. R. M. Norman

*F.R.C.O.G.*

Mr. W. G. MacGregor
Mr. M. P. Embrey

### *UNITED BRISTOL HOSPITALS JANUARY TO JUNE,* 1961

| *Name* | *Appointment* | *Formerly* |
|---|---|---|
| LLOYD, O. C., M.A., M.D., B.Ch. | Consultant Pathologist (Hon. contract) in the United Bristol Hospitals during tenure of the post of Lecturer in Pathology in the University of Bristol. | Lecturer in Pathology, University of Bristol. |
| HADLEY, K. J., B.SC., M.B., Ch.B., F.R.C.S. | Senior Registrar in E.N.T. Surgery | Registrar in Radiotherapy, United Birmingham Hospitals |
| LAIDLAW, C. D'ARCY, M.B., B.S., F.R.C.S. | Senior Surgical Registrar. | Senior Surgical Registrar, Southmead Hospital. |
| DUKE, R. F. N., B.M., B.Ch., F.R.C.S. | Senior Registrar in Orthopaedics. | Registrar General Surgery, Dulwich Hospital. |
| METHA, F. F., B.D.S. | Registrar in Dental Surgery. | S.H.O. in Dental Surgery, Bristol Dental Hospital. |
| DAVIE, J. P., M.B., Ch.B., D.A. | Registrar in Anaesthetics. | Registrar in Anaesthetics, Leicester Royal Infirmary. |
| MOULE, N. J., M.B., Ch.B. | Part-time Registrar in Radiology. | S.H.O. in Surgery, U.B.H. |
| SMITH, J., M.B., Ch.B. | Part-time Registrar in Radiology. | Medical Registrar, Prince of Wales's General Hospital. |
| HENRY, A. N., B.A., M.B., Ch.B., B.A.O., F.R.C.S.I., F.R.C.S. | Registrar in Orthopaedics. | R.S.O., Dartford Group of Hospitals. |
| McCARTHY, C. F., M.B., B.Ch., B.A.O., M.R.C.P. | Registrar in Medicine. | Research Assistant Brompton Hospital. |
| BEVAN, G. H., M.B., B.S. | Registrar in Opthalmology. | S.H.O. in Opthalmics, Moorfield Eye Hospital. |
| BROOKS, A. W., B.D.S., L.D.S.R.C.S. | Registrar in Dental Surgery (Hon. contract) in the United Bristol Hospitals during tenure of the post of Lecturer in Dental Surgery in the University of Bristol. | Lecturer in Dental Surgery, University of Bristol. |
| MORTIMER, K. V., B.D.S., L.D.S.R.C.S. | Registrar in Dental Surgery (Hon. contract) in the United Bristol Hospitals during tenure of the post of Lecturer in Dental surgery in the University. | Lecturer in Dental Surgery, University of Bristol. |

### *SOUTH WESTERN REGIONAL HOSPITAL BOARD*
### *APRIL TO JUNE,* 1961

| *Name* | *Appointment* | *Formerly* |
|---|---|---|
| | *BRISTOL* | |
| Skinner, P. A., M.B., B.S., F.F.A.R.C.S. | Consultant Anaesthetist Frenchay Hospital, Bristol. | Senior Registrar, Frenchay Hospital, Bristol. |
| Basu, S. K., M.B., D.A. | Anaesthtic Registrar, Southmead Hospital, Bristol. | Anaesthetic Redistrar, Taunton and Somerset Hospital, Taunton. |
| Bennetts, F. E., M.B., B.S., D.A., F.F.A.R.C.S. | Senior Anaesthetic Registrar, Frenchay Hospital, Bristol. | Anaesthetic Registrar, Southampton Group of Hospitals. |
| | *BATH* | |
| Crosfill, M. L., M.B., B.S., F.R.C.S. | Surgical Registrar, Bath Group of Hospitals. | Surgical Registrar, Royal Masonic Hospital. |
| Corney, G., M.B., ch.B., D.obst., R.C.O.G., D.C.H. | Medical Registrar, Bath Group of Hospitals. | Paediatric Registrar, Sorrento Maternity, Little Bromwich General and Solihull Hospitals. |
| Voyce, M. A., B.SC., M.B., ch.B. | Medical Registrar, Bath Group of Hospitals. | S.H.O., Bath Group of Hospitals. |
| Ellison, D. J., M.B., ch.B., M.R.C.P. | Clinical Assistants in General Medicine in the Bath Group of Hospitals each undertaking one session weekly. Annual appointments for the year commencing 1.8.61 | Clinical Asst. Bath and in general practice |
| Ronn, H. H., M.B., B.S.' M.R.C.P. | | Clinical Asst. Bath and in general practice. |
| Bazeley, R. W., M.B., B.S. | | In general practice in Radstock. |
| Fladee, H. W., B.SC., M.B., ch.B., D.obst., R.C.O.G. | | In general practice in Castle Combe. |
| Ree, M. J., M.B., ch.B., D.obst., R.C.O.G., D.C.H. | Clinical Assistant in Paediatrics, Royal United Hospital, Bath. (1 session). Annual appt., for one year commencing 1.8.61 | Asst. in general practice at Keynsham. |
| Trafford, P. A., M.B., B.S. | Clinical Assistant in Ophthalmology, Bath Eye Infirmary. (2 sessions). | In general practice in Bruton. |
| | *NORTH GLOUCESTERSHIRE* | |
| Edwards, E. M., M.B., B.S., M.S., M.R.C.O.G. | Consultant Obstetrician and Gynaecologist, North Gloucestershire Clinical Area. | Lecturer in Obstetrics and Gynaecology, University of Bristol. |
| | *SOUTH SOMERSET* | |
| Woo, F. J. Y., M.A., M.D. | Consultant Physician with an interest in Geriatrics, South Somerset Clinical Area. | Senior Medical Registrar, Bristol Royal Hospital, and Tutor in Medicine, University of Bristol. |
| | *DEVON AND EXETER* | |
| Muir, B. J., B.A., M.B., B.chir., D.A., F.F.A.R.C.S. | Consultant Anaesthetist, North Devon Infirmary, Barnstaple. | Senior Registrar, Manchester Royal Infirmary. |
| Grainger, C. N., M.B., B.S., M.D. | Clinical Assistant in Dermatology, Exeter (1 session) re-appointed. | Clinical Asst. in Exeter and is in general practice in Totnes. |

W1 ME 304

### 1961 INDEX TO VOL. 76

### SUBJECTS

| | page |
|---|---|
| Agranulocytosis, osteomyelitis and | 25 |
| Amyloidosis, coagulation defect in | 97 |
| Ankylosing spondylitis | 132 |
| Cave rescue | 37 |
| Cervical disc, the unstable | 57 |
| Clinico-pathological conferences | 25, 65, 132 |
| Clonorchiasis | 65 |
| Fungus infections | 112 |
| General Medical Council | 75 |
| Geriatric services | 12 |
| Headache | 122 |
| Hemiballism | 92 |
| Medical School, The University and | 103 |
| Medical treatment, diseases due to | 1 |
| Notes and news | 35, 71, 101, 143 |
| Osteomyelitis, chronic, and agranulocytosis | 25 |
| Pasteurella multocida | 129 |
| Prostatectomy, indications for | 85 |
| Rectum, restorative surgery | 7 |
| Skeleton in the pit | 61 |
| Subarachnoid haemorrhage | 50 |
| University and Medical School | 103 |

### AUTHORS

| | |
|---|---|
| Brocklehurst, R. J. | 75 |
| Cullen, W. | 92 |
| English, M. P. | 112 |
| Fraser, I. D. | 97 |
| Hayes, G. H. | 129 |
| Hoffman, H. L. | 50 |
| Jones, D. M. | 57 |
| Kersley, G. D. | 20 |
| Leitch, A. | 92 |
| Lloyd, O. C. | 39 |
| Lloyd, T. W. | 12 |
| Mitchell, J. P. | 85 |
| Morris, P. | 103 |
| Neale, G. | 129 |
| Page, F. T. | 122 |
| Perry, C. B. | 1 |
| Sames, C. P. | 7 |
| Smith, G. S. | 61 |
| Wills, M. R. | 97 |

